# Spatial Characteristics of Heavy Metals in Street Dust of Coal Railway Transportation Hubs: A Case Study in Yuanping, China

**DOI:** 10.3390/ijerph15122662

**Published:** 2018-11-27

**Authors:** Dongyue Li, Yilan Liao

**Affiliations:** 1 The State Key Laboratory of Resources and Environmental Information System, Institute of Geographic Sciences and Natural Resources Research, Chinese Academy of Sciences, Beijing 100101, China; lidy@lreis.ac.cn; 2 College of Resources and Environment, University of Chinese Academy of Sciences, Beijing 100049, China; 3 Jiangsu Center for Collaborative Innovation in Geographical Information Resource Development and Application, Nanjing 210023, China

**Keywords:** street dust, heavy metals, coal transportation, spatial distribution

## Abstract

Coal is a vital basic energy source in China, and rail serving is its major mode of transportation. Heavy metals in street dust surrounding the coal railway do harm to the environment and pose a potential risk to human health. This paper aims to identify the effects of coal transportation hubs on heavy metals in street dust. The geoaccumulation index and ecological risk index were used to assess the contamination levels of the following elements in Yuanping, Shanxi: arsenic (As), cadmium (Cd), chromium (Cr), copper (Cu), mercury (Hg), nickel (Ni), lead (Pb), and zinc (Zn). The levels of contamination of these heavy metals in soils were compared to those in street dust, and the difference between the railway’s and mining’s impacts on dust’s heavy-metal concentrations was explored. The results indicated that Cr and Pb in street dust were mainly affected by coal railway transportation, and the interaction effect of coal railway transportation and mining was greater than either of them alone. A potential control and prevention zone for Cr and Pb extending 1 km to both sides of the railway was identified. This work proves that coal railway transportation has certain effect on heavy metals in street dust and provides a scientific approach for future environmental impact assessments of coal transportation via railway.

## 1. Introduction

China is rich in coal resources, and coal remains a basic energy source there [1,2,3], securing it a significant position in the national economy. China’s coal production is mainly located in the northern China centred on Shanxi Province; in the eastern China centred on Shandong Province and Huai River; in south-western China centred on Guizhou Province; and, in north-western and north-eastern China. However, the demand for coal is concentrated in the economically developed, coastal areas of eastern China and the southern China [4,5]. Consequently, an abundance of coal is transported over long distances. At present, it is transported mainly by waterway, highway, and railway, with railway being the major mode of transportation [6] due to its large-volume capacity, ability to cover long distances, high speed, and freedom from interference by climatic conditions. As more coal accumulates in the coal transportation hubs, one expects a higher concentration of heavy metals in the areas surrounding these hubs, which does harm to the environment. Due to vibration, leakage, and the effect of wind [7] in railway transportation, coal dust is introduced into the environment along railway lines and it causes heavy metals to accumulate [8].

Street dust is the accumulation of solid particles on outdoor ground surfaces [9,10]. Street dust affects urban environmental quality, introducing pollution via multiple modes [11,12]. Many studies showed that heavy metals in street dust do not degrade, but rather persist in street dust [13,14,15]. As street dust permeates the ecological environment by many means, such as surface runoff and atmospheric precipitation [16,17,18], heavy metals contained in street dust eventually enter the food chain, which are posing a potential risk to human health [19,20,21,22]. Many recent studies on heavy metals in street dust involved assessments of contamination levels [22,23,24], spatial distribution [11,22,24], and source identification [25,26,27]. Heavy metals in street dust have many sources [28,29,30], which can be divided into two types: natural sources and those based on human activity. Natural sources include soils [31]. Human activity—the main source of heavy metals in street dust—includes vehicle emissions, coal combustion, and building materials. Some studies [32,33] have shown that coal transportation influences the environment, but there are few studies on the effects of coal railway transportation on the presence of heavy metals in street dust. These factors are important in establishing pollution control strategies [34,35], in particular, in coal transportation hubs.

This article describes a study that focused on the coal transportation hub of Yuanping in Shanxi Province and analysed the effects of railway coal transportation on the surrounding environment. The study assessed the levels of contamination in street dust of the following heavy metals: arsenic (As), cadmium (Cd), chromium (Cr), copper (Cu), mercury (Hg), nickel (Ni), lead (Pb), and zinc (Zn). As is a metalloid, but its physicochemical properties are similar to those of heavy metals, so As is usually analysed according to the analysis method of heavy metals [11,36] and the term “heavy metal” was used for all the elements in this study. The study employed the geoaccumulation index (I_geo_) and ecological risk index (RI; [37,38,39,40]. The relative levels of contamination of heavy metals in soils and in street dust were compared to exclude the influence of soils on street dust contamination. Furthermore, the contamination impacts of mining and transportation were separated by correlation and geographical detector analysis.

## 2. Materials and Methods

### 2.1. Study Area

**Figure 1:**
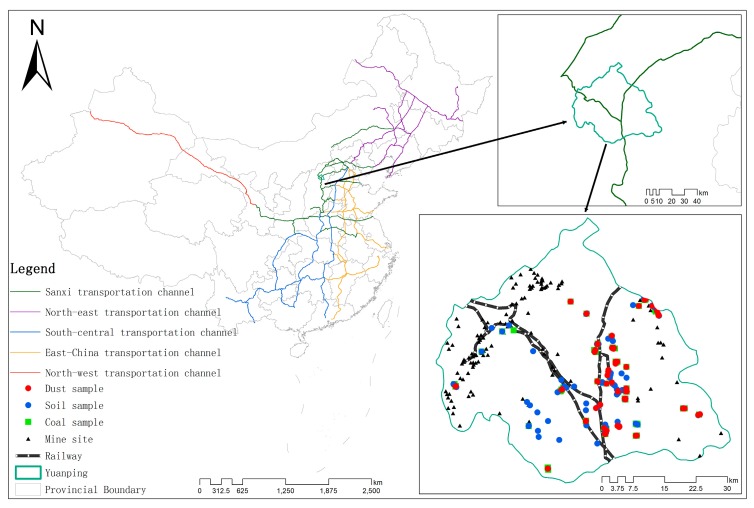
Distribution of street dust and soils sampling locations in Yuanping.

Yuanping (38°35′–39°09′ N, 112°17′–113°35′ E) is located in the north of Shanxi Province and a major coal transportation hub between the south of Shanxi and northern China (Figure 1). The city has an area of approximately 2560 km^2^ and is rich in mineral resources. It is surrounded by many large coalfields distributed in the northwest: Ningwu, Datong, Xishan, and Qinshui. Yuanping has a prevailing northwest wind that occurs in spring. It belongs to the temperate continental climate zone and has an average annual temperature of 9.8 °C and average annual precipitation of 417.1 mL. Yuanping is located along the Sanxi coal transportation channel (contains coal transportation channels in Shanxi, Shaanxi, and western Inner Mongolia), and the Beijing–Yuanping, Beitongpu, and Shuohuang railways intersect in the city, which has a total transportation area of 39.87 km^2^.

### 2.2. Sampling and Chemical Analysis

Based on stratified, multi-phased, and cluster probability statistical methods, a total of 432 villages were sampled in the 520 villages in Yuanping. In the same way, 51 of the most important villages were further sampled form the 432 villages. Two samples were sampled per village, after removing invalid, contaminated samples, 94 street dust samples, 93 topsoil samples, and 77 coal samples were collected. The geographic coordinates of all sampling points were recorded using a global positioning system (GPS). All samples were kept in polyethylene bags and brought back to the laboratory after marking. The samples were collected in the following manner: Firstly, for street dust samples (Figure 1), at each street dust sampling site, a street dust sample of approximately 200 g was swept into a polyethylene bag with a brush by gently sweeping an area of about 1 m^2^ at the side of the road. Secondly, for topsoil samples (Figure 1), five subsamples were collected with a stainless-steel auger at each topsoil sampling site within 10 m^2^ and were mixed into one composite sample. Thirdly, for coal samples (Figure 1), sampling sites were mainly located at surrounding coal temporary storage points, coal washing pools, and household coal storage locations along the railway lines, four subsamples in four directions and one subsample on the top of a coal stack were collected at the temporary and household storage sites, four subsamples in four corners of each coal washing pool were collected. Subsamples that were collected at a given sampling site were mixed into one composite sample.

All samples were processed uniformly. After air drying, manually removing plant materials and pebbles, and finally, grinding, all of the samples were sieved through a 1-mm mesh nylon sieve. The concentrations of As, Cd, Cr, Cu, Hg, Ni, Pb, and Zn in street dust, topsoil, or coal were measured. Samples of 5 g each were pressed into thin slices using a tablet machine, As and Zn concentrations were measured using X-ray fluorescence spectrometry. A sample of about 0.5 g and a small amount of water were mixed into a beaker, then the samples were heated to near dry after adding 10 mL hydrochloric acid (HCl, 36%), finally Cd concentration was measured using inductively coupled plasma mass spectrometry (ICP-MS) [41]. A sample of about 0.1 g and a small amount of water were mixed into a Teflon crucible, 3 mL nitric acid (HNO_3_, 69%), 1 mL hydrofluoric acid (HF, 40%), 1 mL perchloric acid (HClO_4_, 70%), and 2 mL HCl (36%) were added, after complete digestion and evaporation, Cr, Cu, Ni, and Pb concentrations were measured using inductively coupled plasma optical emission spectrometry (ICP-OES). A sample of about 0.5 g and a small amount of water was added into a digester, 10 mL of a mix of HNO_3_ (69%), and sulfuric acid (H_2_SO_4_, 98%), as well as 10 mL potassium permanganate (KMnO_4_, 2%), were added to dissolve Hg was determined its concentration by flame atomic absorption spectrometry (F-AAS, Beijing, China; [42,43]). The data analysis of this study was completed using SPSS 22.0 software (New York, NY, USA).

### 2.3. Contamination Assessment Methods for Heavy Metals

#### 2.3.1. Geoaccumulation Index ($I_{geo}$)

The $I_{geo}$, introduced by Müller [44], is the most popular index used to evaluate pollution [39,45] and it takes both the background value and diagenesis into consideration [44,46]. $I_{geo}$ is defined by the following equation:

(1) $$I_{geo}=\log_{2}(C_{i}/1.5C_{bi})$$

where $C_{i}$ is measured concentration of element i and $C_{bi}$ is geochemical background reference value of element i. In this study, $C_{bi}$ is taken from the national primary standard for heavy metals in soils in China (Table 1). The constant of 1.5 is the correction factor for lithological actions. The following classification scale is given for $I_{geo}$ [23,37]: $I_{geo}$ ≤ 0 is classified as *practically unpolluted*; 0 < $I_{geo}$ ≤ 1, *unpolluted to moderately polluted*; 1 < $I_{geo}$ ≤ 2, *moderately polluted*; 2 < $I_{geo}$ ≤ 3, *moderately to strongly polluted*; 3 < $I_{geo}$ ≤ 4, *strongly polluted*; 4 < $I_{geo}$ ≤ 5, *strongly to extremely polluted*; and, $I_{geo}$ > 5, extremely polluted.

#### 2.3.2. Potential Ecological Risk Index (RI)

The RI was originally introduced by Hakanson [47] and quantitatively expresses the potential ecological risk of single or multiple elements. The RI is calculated as follows [39,47]:

(2) $$\mathrm{RI}=\sum_{i=1}^{n}E_{r},$$

(3) $$E_{r}=T_{r}\times C_{f},$$

(4) $$C_{f}=\frac{C_{s}}{C_{n}},$$

where $E_{r}$ is the ecological risk factor of Element x and $T_{r}$ is the toxic response factor of Element x, known for As, Cd, Cr, Cu, Hg, Ni, Pb, and Zn to be 10, 30, 2, 5, 40, 5, 5, and 1, respectively [48,49]. $C_{s}$ and $C_{n}$ are the content of element and geochemical background reference values [50], respectively. The following classification scheme is used to describe risk levels: $E_{r}$ < 40 is classified as low potential ecological risk; 40 ≤ $E_{r}$ < 80, moderate potential ecological risk; 80 ≤ $E_{r}$ < 160, considerable potential ecological risk; 160 ≤ $E_{r}$ < 320, high potential ecological risk; and, $E_{r}$ ≥ 320, extreme ecological risk. Moreover, RI < 150 is classified as low ecological risk; 150 ≤ RI < 300, moderate ecological risk; 300 ≤ RI < 600, considerable ecological risk; and, RI ≥ 600, extreme ecological risk. Contamination assessment was processed using SPSS 22.0 software, and the distribution of heavy metals in street dust was mapped using ArcGIS 10.4 software (Environmental Systems Research Institute, Redlands, CA, USA).

### 2.4. Geographical Detector

Geographical detector methodology is often used to explore the influence of geospatial factors on disease risk [51]. The geographical detector is grounded on the power of determinant (PD), which generates four detectors [51,52]: risk detector, factor detector, ecological detector, and interaction detector. The risk detector is used to explore the main risk region; the factor detector is used to identify factors that caused said risk. The ecological detector is mainly used to explain the relative importance of risk factors, and the interactive detector can be used to explain whether the impact factor was independent or interactive [51,52]. The biggest advantage of the geographical detector is that there are no assumptions that effectively overcome the limitations of dealing with class variables in traditional statistical analysis methods.

(5) $$\mathrm{PD}=1-\frac{1}{N\sigma^{2}}\sum_{i=1}^{L}N_{i}{\sigma_{i}}^{2}$$

where N and $\sigma^{2}$ denote the number and variance, respectively, of measured element concentrations in a study area and $N_{i}$ and $\sigma_{i}$ denote the number and variance, respectively, of measured element concentrations in stratum i (i=1,2,…,L) [53]. The PD gives the impact degree of a factor on the research object, namely the impact of distances to the railway and mines on the element content in this study. The PD value is between 0 and 1; a high PD value indicates a high effect of the factor on the research object. The geographical detector process was performed using Geographical Detector 2015 software [51].

### 2.5. Assessment Procedure

**Figure 2:**
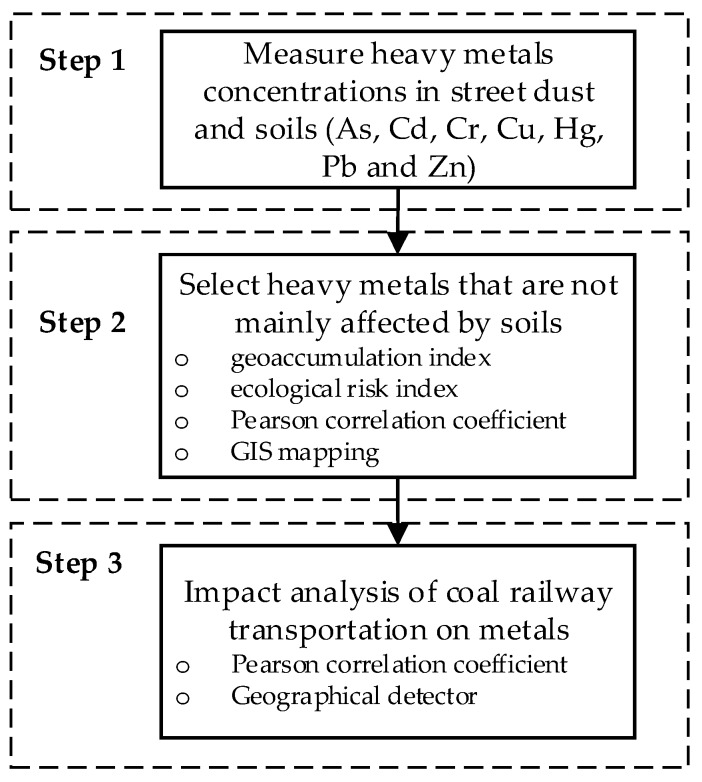
Process of accessing spatial characteristics of heavy metals in street dust of coal railway transportation hubs.

The procedure for assessing spatial interpolation in estimating heavy metals in soils in this study can be described using three main steps (Figure 2):

Step 1 Concentrations of heavy metals in street dust and soils sampling data were measured.

Step 2 Geoaccumulation index, ecological risk index, Pearson correlation coefficient, and geographic information system (GIS) mapping methods were used to analyze the effect of soils on the heavy-metal levels and selected heavy metals that were not mainly affected by soils.

Step 3 Pearson correlation coefficient and Geographical detector were used to analyze the impact of coal railway transportation on heavy metals in street dust.

## 3. Results

### 3.1. General Statistics of Heavy Metals

The national primary standard for heavy metals in soils in China (Environmental Quality Standard GB 15618-1995) is also used for analysis heavy metals in street dust [11,28,38], so it is used as background value for street dust and soils in this study. Previous studies have shown that several heavy metals in street dust may be affected by the local soils [38,43]. For this reason, the descriptive statistics of heavy metals in street dust and soils were provided in Table 1. Among them, Pb, Zn, and Cr stood out with larger concentration value ranges. The national primary standard (NPS) for heavy metals in soils in China (GB 15618–1995) was used as the background value. The mean concentrations in street dust of all the heavy metals, except As, Cd, and Ni exceeded their corresponding background values. Among them, the mean concentration of Pb was the highest at 13 times that of its background value.

The minimum concentrations in street dust of all the elements exceeded those in soils, except As, Cd, and Cr. Similarly, the maximum concentrations in street dust of all elements exceeded those in soils, except As, and the mean concentrations in street dust of all except As and Cd exceeded those in soils. Moreover, the percentage of polluted sites out of the total number of sampling points was greater for street dust than for soils. These findings indicated that the concentrations of most heavy metals in street dust (excluding As) were far beyond those found in soils, and heavy metals in street dust might be affected by other factors.

### 3.2. Contamination Level Assessment

#### 3.2.1. Contamination Level Analysis

**Figure 3:**
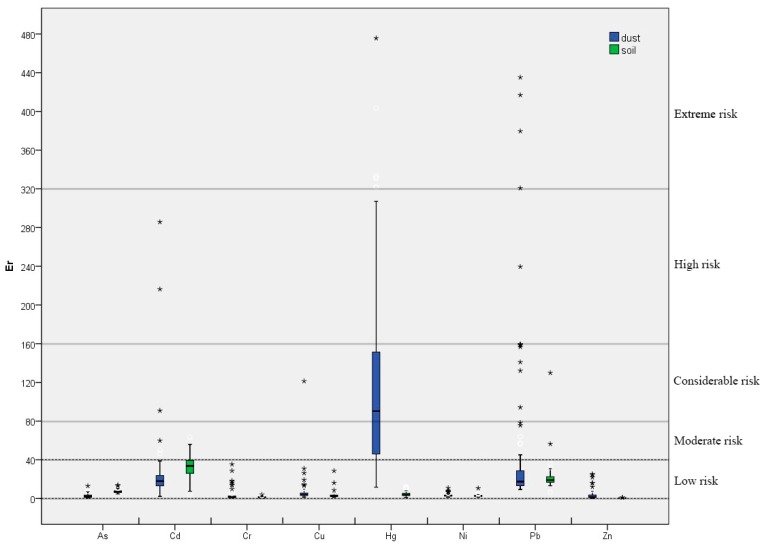
Box-plots of $I_{geo}$ for heavy metals.

The $I_{geo}$ values for the heavy metals in street dust and soils were presented in Figure 3. In street dust, none of the sampling points were polluted by As, and most of the sampling points were not polluted by Cd, Cr, Cu, or Ni. For Hg and Zn. Most points had $I_{geo}$ values between 0 and 1, indicating that the samples were unpolluted or had moderate pollution levels. A small number of sampling points for Hg and Zn had $I_{geo}$ values between 1 and 2, indicating moderate pollution. For Pb, most sampling points also had $I_{geo}$ values between 1 and 2, or moderate pollution. 

According to mean $I_{geo}$ values, the pollution levels in street dust were higher than those in soils in the cases of Pb, Hg, Zn, Cr, and Cu, but they were lower in the case of Cd. As and Ni did not cause any pollution in street dust. In other words, the sampled street dust was contaminated to some extent ($I_{geo}$ > 0) by heavy metals, such as Hg, Zn, Cr, Cu, and particularly by Pb. The sampled soils contained some level of Pb, Cd and Cr pollution ($I_{geo}$ > 0), indicating that the initial source of some heavy metals in street dust was different from that of soils [31].

#### 3.2.2. Ecological Risk Analysis

**Figure 4:**
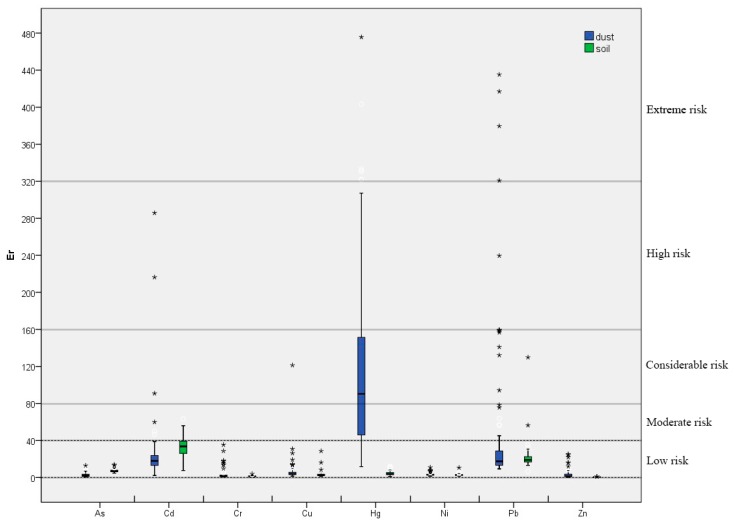
Box-plots of $E_{r}$ for heavy metals.

The $E_{r}$ results for the heavy metals in street dust and soils were shown in Figure 4. For street dust, almost all sampling points reported low potential ecological risk from As, Cd, Cr, Cu, Ni, Pb, and Zn. Most of the $E_{r}$ values of Hg were between 40 and 160, indicating moderate to considerable potential ecological risk, while a few sampling points showed a similar moderate-to-considerable potential risk from Cd, Cu, and Pb. A small number of sampling points returned $E_{r}$ results, which indicated a high potential for ecological risk from Cd and Pb and an extremely high risk from Hg and Pb. On the whole, the potential ecological risk results of street dust samples were significantly higher than those of soils samples, particularly in the cases of Cr, Cu, Hg, Pb, and Zn.

**Figure 5:**
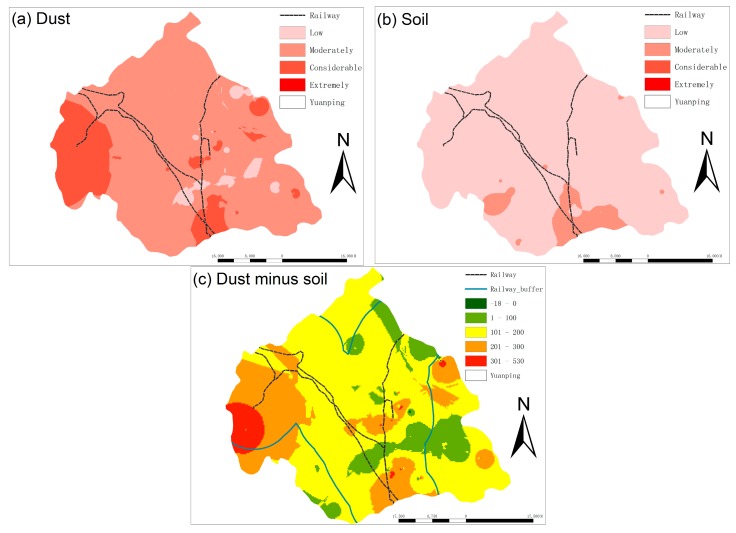
Spatial distribution of RI in (**a**) street dust, (**b**) soils, and (**c**) street dust minus soils.

The RI for the maximum, minimum and mean $E_{r}$ for each heavy metal in soils and street dust were shown in Table 2. The RI values in street dust were higher than those in soils, indicating that the ecological risk presented by street dust is more serious than that of soils. The distributions of RI in street dust and soils by ordinary kriging were shown in Figure 5 to improve understanding by showing the ecological risk in spatial terms. The RI values of street dust were higher than 150 in most areas, indicating moderate ecological risk, while the findings showed that some areas in western, central, and south-central Yuanping face considerable risk, with RI values greater than 300. Certain individual sites had RI values greater than 600 and thus face extreme ecological risks. Figure 5a showed that high RI values in street dust were mostly found in areas surrounding railway lines. Meanwhile, the RI values of soils in most areas indicated low ecological risk, although some areas in the south of Yuanping face moderate ecological risk (Figure 5b).

The differences in ecological risk that are presented by street dust and soils were computed using RI values of street dust samples minus those of soils (Figure 5c). The differences were larger in the north-western, central, south-central, and north-eastern regions of Yuanping, which are areas generally distributed around the railway (Figure 5c). In almost all areas, the RI values of heavy metals were higher in street dust than in soils. In other words, a greater ecological risk is posed by heavy metals in street dust than in soils. The impact of street dust has been shown to be contained within 10 km of railway lines [54], as illustrated on the map in Figure 5c by a 10-km zone on either side of the railway. Most of the high-value regions were within this zone, indicating that the railway might have caused the different concentrations in heavy metals between street dust and soils.

### 3.3. Distribution of Heavy-Metal Concentrations

In order to obtain the most accurate distribution possible using the available land-use areal data, one new and two commonly used methods were tested using interpolation of the Cr data. The commonly used methods were ordinary kriging (OK) and inverse distance weighted interpolation (IDW), and the new method was area-and-point kriging (AAPK). For Cr, 70% of the sampling data was used for interpolation and the remaining 30% was used for verification. The mean squared error of the OK, IDW, and AAPK methods were 197.3 mg/kg, 250.6 mg/kg, and 189.8 mg/kg, respectively. Consequently, AAPK was used to find the distribution of heavy metals in street dust and soils.

**Figure 6:**
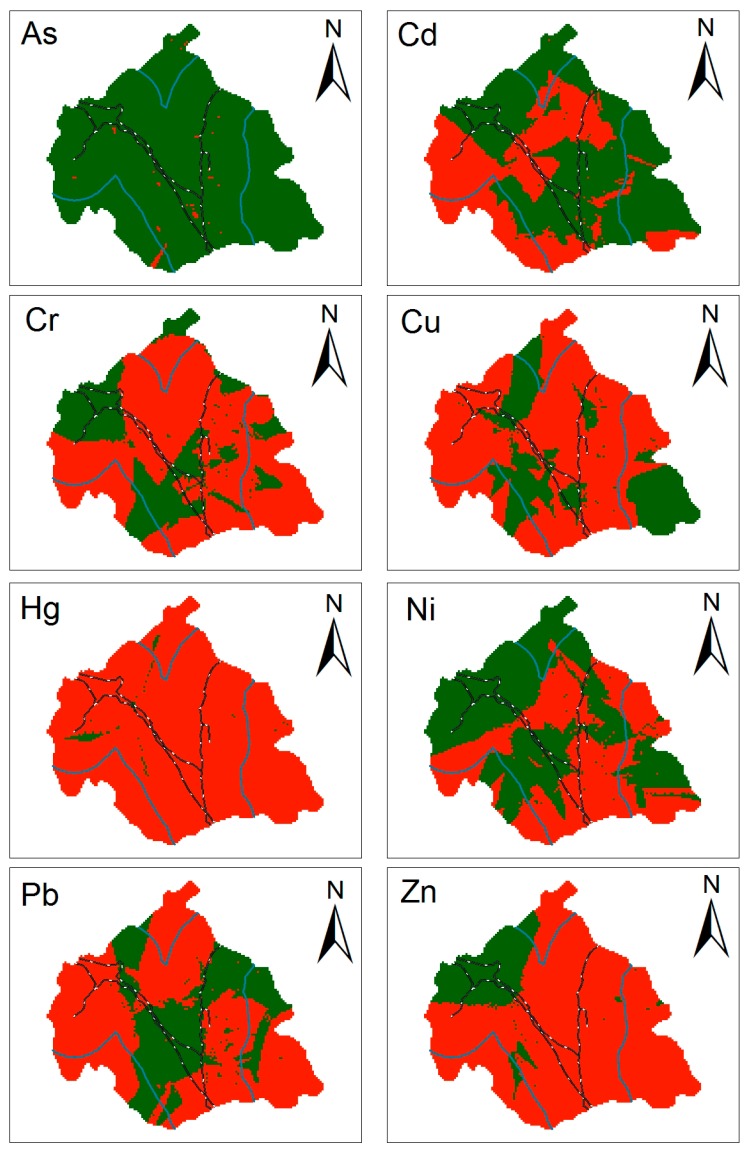
Spatial distribution of heavy-metal concentrations in street dust minus that in soils (green (darker areas on the map) means negative; red (lighter) means positive; blue (solid) lines indicate the 10-km zone surrounding the railway lines; black and white (dotted) lines show the railway lines; unit: mg/kg).

The differences in the concentrations of heavy metals in street dust and soils were shown in Figure 6. The red area represented regions where the heavy-metal content in street dust was higher than that in soils, and the green areas showed where the heavy-metal content in street dust was lower than that in soils. Except for As, the concentrations of the heavy metals in street dust were higher than those in soils to varying extents. In particular, the concentrations of Cr, Cu, Hg, Pb, and Zn in street dust were generally higher than that in soils. Cr, Cu, Hg, Pb, and Zn in street dust must be affected by other factors [55,56]. Even more noticeable was the fact that the Hg content in street dust was greater than that in soils almost everywhere. The maps in Figure 6 identified a roughly 10-km buffer zone surrounding the railway lines [54]. Most of the red regions of all eight elements fell within the buffer zone, indicating once again that the railway may be the reason for the difference in the heavy-metal content of street dust and soils [57,58].

### 3.4. Impact Analysis of the Coal Transportation Channel on Heavy-Metal Content

From the above analysis, it was assumed that the railway had some influence on the heavy-metal content of street dust, especially for Cr, Cu, Hg, Pb, and Zn. Since the railway’s effect on street dust was confined to an area within 10 km of the track, those sampling points of the five heavy metals that fell within this area (80 of the total 94) were selected to analyse the correlation between the distance to the railway and the heavy-metal concentrations in the sampled street dust. The correlation analysis of this study was completed using R software (The University of Auckland, Auckland, New Zealand).

**Figure 7:**
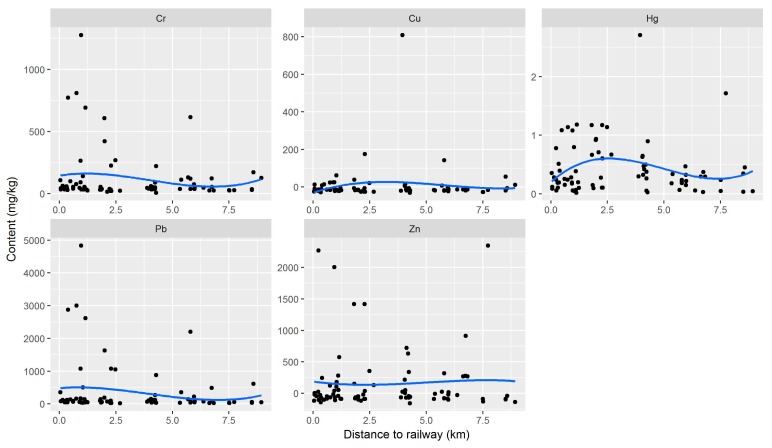
Relationship between differences in heavy-metal content (between street dust and transported-coal) and distances from street dust collection sites to the railway.

To further illustrate the similarity of the heavy-metal content in street dust and coal transported on the railway, this study analysed the relationship between differences in the heavy-metal content and the distances from street dust collection sites to the railway (Figure 7). Because the heavy-metal concentrations of transported coal on the railway was unavailable to test, coal samples near the railway were used instead. Transported coal is loaded and unloaded mainly in the 1.5 km around the railway [54]; the coal samples near the railway (24/77 coal samples within 1.5 km around the railway) were very similar to the coal transported on the railway. Therefore, the maximum value of heavy-metal concentrations for the 24 samples was selected as the heavy-metal content of coal transported on the railway (Table 3). The heavy-metal content of street dust was predicted to be lower than that of the coal transported on the railway, with the difference—expected to be less than 0—increasing as the distance between the street dust sample site and the railway increased. However, there were many high content sampling points, indicating that other factors, such as nearby coal mines, had affected the heavy-metal concentrations in street dust. Nevertheless, the differences in concentrations of Cr and Pb in street dust and transported coal increased slightly as the distance between the sample site and the railway increased, up to about 7 km for Cr and Pb. These findings indicated that Cr and Pb concentrations in street dust were affected by the railway to some extent.

**Figure 8:**
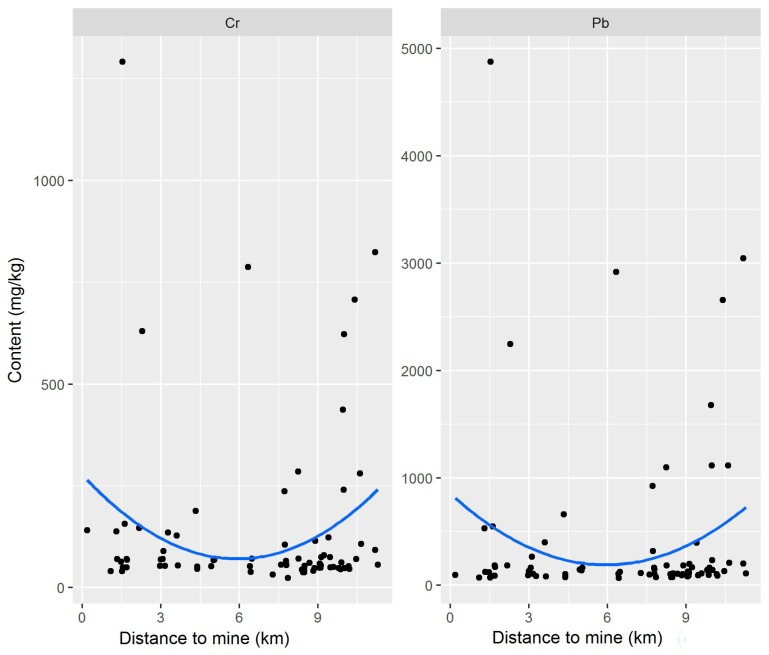
Relationship between the heavy-metal content of street dust and distance of sample sites to mines.

The relationship between the heavy-metal content of street dust and distance to mines is shown in Figure 8. The mines in Yuanping are mainly coal mines. There are 121 major mines in Yuanping, including 98 coal mines, 12 iron ore mines, and 11 quartzite mines. Some values of heavy metals in street dust were higher than that in coal transported on the railway, which may be explained by the presence of mines. Figure 8 showed that, as distance increased between the mines and street dust collection sites, the heavy-metal levels first decreased and then increased. 

The Pearson correlation coefficient was used to measure the degree to which distances to the railway and to mines impacted heavy-metal content in street dust samples. The results of this analysis were shown in the Before Segment column in Table 4. The correlation coefficients for the distance to the railway of only two heavy metals, Cr and Pb, were negative, but the results were insignificant, with *p*-values of 0.142 and 0.121, respectively. The results were weaker for mines. As such, the correlations were not significant, which is likely due to the bias distribution of street dust sampling points (Figure 1). In order to address the bias, 80 sampling points were segmented, with the goal of an equal number of sampling points per segment. The Pearson correlation of the mean heavy-metal concentration in each section and the mean of the corresponding distances to the railway lines and mines were then calculated. The results were shown in the After Segment column in Table 4. Using three or four segments achieved high Pearson correlation coefficient results and significant p-values for all three heavy metals—better results than were achieved with other segmentations. However, the number of sampling points was different in each segment. The best unbiased results were achieved for Cr and Pb for the railway, which had almost the same number of samples in each segment. The sample bias was the greatest for mines with the highest sample bias of 38 for Cr. Pearson correlation analysis showed that both coal transportation and local coal mining had stronger effects on the heavy-metal content of street dust after renewing the spatial scale [59,60] than before.

The geographical detector method was then used to measure the degree to which the distances to the railway and mines impacted heavy-metal content in street dust. The PD results were shown in Table 5. The PDs of railway distance on heavy-metal concentrations were in the following order: Pb > Cr. However, the PDs of mine distance on heavy-metal concentrations were in a different order: Cr > Pb. It should be noted that the PDs of railway distance on heavy-metal concentrations were greater than that of mine distance, indicating that the effect of railway transportation on heavy metal levels in street dust is greater than that of mining production. In other words, Cr and Pb in street dust were influenced more by coal transported via the railway than by coal in local mines. In addition, the interaction between railway and mines enhanced each other (Table 5), as the PDs were in the following order after interaction: Cr > Pb. The railway-mines interaction enhanced their individual influences on heavy-metal levels in street dust (Table 5).

Of the segments studied, the one closest to the railway had the highest levels of heavy metals in street dust, followed by the mid-distance and the farthest segments. For Cr and Pb, the first boundary occurred about 1 km from the railway (as shown in Table 6). This finding supported the idea that the government should focus primarily on these zones when taking actions to protect human health and the environment; for example, the government should focus on these zones when allocating funds for clean up. The second segment was 1–4 km from the railway for Cr and 1–2.5 km for Pb. These zones should be monitored by the government.

## 4. Discussion

By identifying the impact of the coal transportation railway in Yuanping on the heavy-metal pollution in street dust, the results demonstrated that Cr and Pb concentrations in street dust were affected by the railway and the impact of coal transportation via railway on heavy metals in surrounding areas is a significant concern. In this article, the results from the combined application of contamination assessment methods, correlation analysis, and GIS methods were acceptable and this analysis frame could be applied to analyse the impact of coal transportation on heavy metals in street dust.

**Figure 9:**
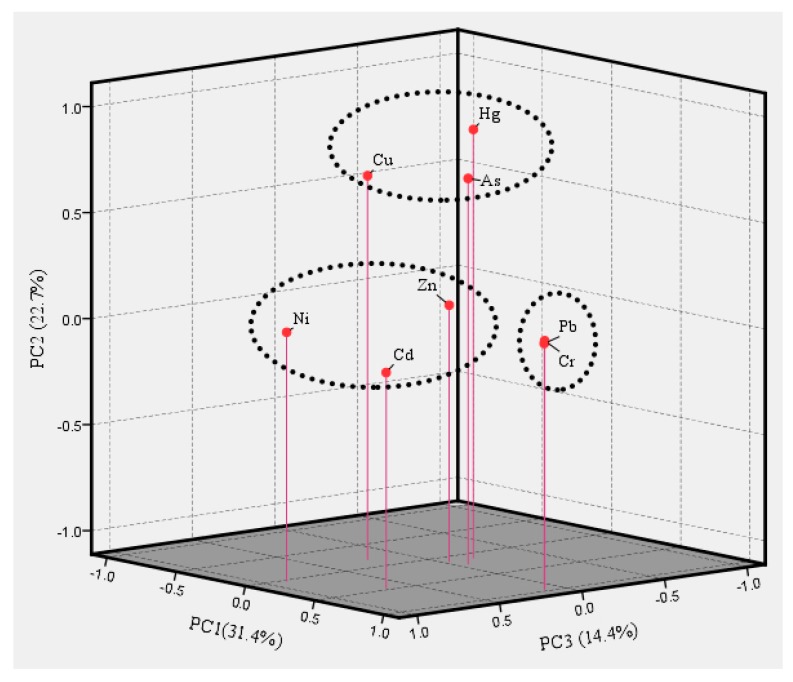
Three-dimensional (3D)-plot of the first three components according to the PCA results. PCA: principal component analysis.

Heavy metals in street dust mainly originate from natural sources [11,29,30], such as soils [31] and human activity, such as mining. Transportation networks are the main source of street dust and the main contributors to heavy metals in street dust in some developed transportation areas [43]. The principal component analysis (PCA) is a generally used method to identify the pollution source the heavy metals in street dust [28]. The Bartlett’s sphericity test was significant at *p* < 0.001, which confirmed that the heavy-metal concentrations in street dust were suitable for PCA in this study. The first principle component (PC1) explains 31.4% of the total variance and included significant loadings for Cr and Pb with loading values of 0.97 and 0.96, respectively (Figure 9), indicating that they have a same potential source of pollution. Of course, heavy metals in soils could transfer to street dust [31], but concentrations and ecological risk of Cr and Pb were higher in street dust than that in soils, suggesting that the pollution of these metals resulting from soils is limited. The PD and correlation coefficients results further illustrates that their source was railway and mines, and the effect of railway in street dust was greater than that of mines on Cr and Pb levels in street dust. Cr and Pb are commonly used in different plastics and building materials [28,61]. However, traffic sources is another source of Cr and Pb in street dust [28]. Cr is often generated from coal [27] and Pb is usually found in vehicles [62,63], and coal railway transportation is happens to be the combination of coal and transportation. The second principle component (PC2) explains 22.7% of the total variance, including As, Cu, and Hg (Figure 9). For PC2, As is associated with soil parent materials [64], which can explain why the content of As in soils was higher than that in street dust. Cu is key component of building materials [65,66] and Hg is widely used in pesticides, such as thermometers [67,68,69]. Coal combustion is one of the main sources of Hg in Shanxi province [36,70]. The source of PC2 is mainly related to human life activities. The third principle component (PC3) explains 14.4% of the total variance, including Cd, Ni, and Zn (Figure 9). The enrichment of Cd and Zn in soils was closely correlated to agricultural production [71]. Cd is usually considered as an element included in the use of phosphate fertilizer, livestock manure, and so on [72]. In addition, Cd is an important element of lubricating oil and tires [25]. Ni and Zn can also be found in automobiles [62]. So, the origin of this principle component was mainly related to agricultural activities and vehicular transport [36].

Representative studies that are consistent with the conclusions of this study are presented in Table 7. These studies showed that railways have significant influence on heavy-metal concentrations in surrounding areas [73,74], and the concentrations of several heavy metals decreased with increasing distance from the railroad [75,76]. In coal transportation hubs, the flow of trains through the hubs is resulting in inestimable pollution [77,78]. Because trace Cr and Pb affect human metabolism and may lead to cancer [19,79,80], the government should monitor railway lines [77] for these three heavy metals. A reduction in and better management of coal transportation would benefit the environment surrounding the railway [43]. However, transportation volume is unlikely to decrease given China’s rapid economic development [81].

Except for the amount of transported coal cannot be reduced, many technologies are also lacking to effectively control pollution resulting from coal transportation [77,83] and mining [84], so heavy-metal pollution is inevitable where these activities take place [21,28,85]. In addition, the concentration of heavy metals in coal varies by location due to differences in the geological environment [86]. In Shanxi Province, there have been five coal-forming periods: Late Carboniferous, Early Permian, Middle Jurassic, Tertiary, and Quaternary [87]. Cr and Pb are mainly present in Tertiary brown coals [87]. Tertiary brown coals occur in Yuanqu County and Fanci County, and Fanci County is very close to Yuanping. In Shanxi Province, Late Carboniferous coals occur in the Hedong, Datong, Ningwu, and Xishan coalfields, as well as others [87], some of which are located near Yuanping. New methods to reduce the amount of coal dust that is produced in transportation should be adopted instead [7,88,89], such as covering the freight with tarpaulins or sprinkling water on the coal. Using dustproof partitions and establishing isolation belts around the railway may also be effective in reducing heavy-metal contamination. Mining activity’s contribution to the presence of heavy metals in street dust should also be addressed [84,90], and protective measures should be taken to reduce this contribution [91,92]. 

China relies heavily on coal; as such, there are many coal transportation hubs in the country. The impact of coal transportation hubs on the environment cannot be ignored. The methods that were used in this study are generally applicable to assess any railway’s scope of influence [77,83,93] on heavy metals in street dust at any coal transportation hub. It should be noted, however, that restricting such analyses to the areas immediately around railway lines is insufficient to address the problem of heavy-metal contamination, because contamination is also attributable to other industries and to the population living near the hub [55,56]. These sources of heavy metals should also be identified and addressed. Street dust carried into the study area by wind may also influence the heavy-metal content of local street dust; this “wind-delivered dust” is a factor to be considered in future research.

## 5. Conclusions

In this study, the spatial characteristics of heavy metals in street dust were analysed, as was the impact of the railway in Yuanping, a city in Shanxi, China, on heavy metals in street dust. The results showed that Cr and Pb were mainly affected by coal railway transportation, and the railway–mines interaction effect was stronger than either of the factors acting alone. The government should place a high priority on the zone extending 1 km on either side of the railway for the control and prevention of Cr and Pb contaminations. Mid-distance zones should be monitored by the local government and are defined as follows: 1–4 km from the railway for Cr and 1–2.5 km from the railway for Pb. Our research indicated that coal transportation via railway contributes to heavy-metal pollution in street dust in coal transportation hubs. The combined application of contamination assessment methods, correlation analysis, and GIS methods provides a proper and precise theoretical framework to identify the effects on the environment of coal transportation via railway. The correct interpretation of the impact of coal railways on street dust data would be helpful in environmental source impact assessments. However, this paper just proposed a simple framework to measure the impact of coal railway transportation on heavy-metal contents in street dust, more finer scale research needs to be done in the future, such as how does the coal railway transportation affect heavy metal content, etc.

## Figures and Tables

**Table 1:** Summary statistics of heavy-metal concentrations in soils and street dust (unit: mg/kg).

Element	Source	Mean	Min	Max	STD ^a^	NPS ^b^	Polluted Sites and Percentage (>NPS)
As	dust	4.2	0.7	19.4	3.2	15	2 (2.1%)
	soils	10.9	7.0	20.9	2.3		6 (6.5%)
Cd	dust	0.17	0.00	1.91	0.24	0.2	13 (13.8%)
	soils	0.22	0.03	0.42	0.07		51 (54.8%)
Cr	dust	148.8	23.5	1592.4	248.6	90	28 (28.8%)
	soils	66.1	16.1	182.2	26.7		16 (17.2%)
Cu	dust	46.1	10.4	849.2	89.3	35	26 (27.7%)
	soils	24.0	8.5	200.5	22.8		10 (10.8%)
Hg	dust	0.47	0.04	2.78	0.43	0.15	68 (72.3%)
	soils	0.02	0.01	0.05	0.01		0 (0)
Ni	dust	26.8	11.1	87.6	11.4	40	9 (9.6%)
	soils	23.0	7.4	85.2	8.7		2 (2.2%)
Pb	dust	449.0	65.5	6349.2	964.2	35	94 (100%)
	soils	147.6	45.8	908.8	89.3		93 (100%)
Zn	dust	347.7	28. 6	2529.4	487.7	100	70 (74.5%)
	soils	47.5	20.0	110.3	17.3		1 (1.1%)

^a^ STD denotes standard deviation; ^b^ NPS denotes the national primary standard for heavy metals in soils in China (Environmental Quality Standard GB 15618-1995).

**Table 2:** $E_{r}$ and RI values for measured heavy metals in street dust and soils samples.

Element		E_r_								RI	Ecological Risk
		As	Cd	Cr	Cu	Hg	Ni	Pb	Zn		
**Max**	Dust	13.0	285.7	35.4	121.3	740.1	11.0	907.0	25.3	2138.8	Very high
	Soils	14.0	63.4	4.1	28.6	11.9	10.7	129.8	1.1	263.5	Moderate
**Min**	Dust	0.5	2.1	0.5	1.5	11.8	1.4	9.4	0.3	27.4	Low
	Soils	4.7	3.7	0.4	1.2	1.1	0.9	6.6	0.2	18.7	Low
**Mean**	Dust	2.8	24.8	3.3	6.6	124.1	3.4	64.1	3.5	232.6	Moderate
	Soils	7.2	32.5	1.5	3.4	4.3	2.9	21.1	0.5	73.4	Low

**Table 3:** Estimated heavy-metals concentrations of the coal (unit: mg/kg).

Element	Cr	Cu	Hg	Pb	Zn
**Value**	15.5	41.1	0.07	43.3	184.4

**Table 4:** Correlation coefficients among Cr and Pb concentrations with distance to railway and mines.

Element	Type	Before Segment		After Segment					
		Pearson’s *r*	*p* Value	Number of Segments	Segmented Values (km)	Number of Samples per Segment	Sample Bias ^a^	Pearson’s *r*	*p* Value
**Cr**	railway	−0.166	0.142	4	1.022; 3.946; 6.376	22; 23; 22; 13	10	−0.959	0.041
	mines	−0.008	0.944	3	3.275; 6.435	20; 11; 49	38	−0.998	0.045
**Pb**	railway	−0.175	0.121	4	1.110; 2.482; 5.972	25; 17; 22; 16	9	−0.984	0.015
	mines	−0.010	0.930	3	4.931; 8.395	26; 17; 37	20	−0.997	0.049

^a^ Sample bias: maximum number of samples in a segment minus minimum number of samples in a segment.

**Table 5:** Degree of impact of distance to railway and mines on Cr and Pb concentrations in street dust.

Element	PD		
	Distance to Railway	Distance to Mines	Interaction
**Cr**	0.04	0.01	0.25
**Pb**	0.04	0.00	0.13

**Table 6:** Zones surrounding railway lines requiring government involvement.

Element	First Two Boundaries (km)	Control and Prevention Zone (km)	Monitor Zone (km)
**Cr**	1.02; 3.95	<1	1–4
**Pb**	1.11; 2.48	<1	1–2.5

**Table 7:** Representative studies similar to this study, focusing on railway effects on the surrounding heavy-metal concentrations (*n*: number of samples).

Study Area	Main Conclusion	Reference
Delhi–Ulan section of the Qinghai–Tibet railway in China (*n* = 225)	“Significantly negative correlations between distance and the concentrations of Cu, Zn, Cd, and Pb … Pb, Cd, and Zn in soils were concluded to be influenced by railway.”	[75]
Qinghai–Tibet railway in China (*n* = 127)	“Concentrations of Zn, Cd and Pb were the most affected … by railway transport.”	[73]
Zhengzhou-Putian Section of Longxi-Haizhou Railroad in China (*n* = 20)	“Railroad transportation had tremendous impacts on railroad-side soils … concentrations of Pb, Zn, and Cd in soils … decreased quickly with the distance from the railroad, increased again and formed a secondary peak at certain distances from the railroad, and then gradually decreased with the increase of distance.”	[76]
Chengdu-Kunming railway in China (*n* = 57)	“The concentrations of Cu, Mn, Pb, Cd and Zn decreased with increasing distance from the railroad.”	[82]
One railway junction in northern Poland (*n* = 15)	“The heavy metal contamination level is much higher in the area of the … the railway siding.”	[74]
